# Dapsone Alters Phenotypical and Functional Properties of Human Neutrophils In Vitro

**DOI:** 10.3390/molecules30010113

**Published:** 2024-12-30

**Authors:** Sara Rakočević, Vanja Mališ, Ljiljana Kozić, Anđela Dubovina, Marija Drakul, Dejan Bokonjić, Miodrag Čolić, Dušan Mihajlović

**Affiliations:** 1 Faculty of Medicine Foča, University of East Sarajevo, Studentska 5, 73 300 Foča, Bosnia and Herzegovina; saradrakocevic@gmail.com (S.R.); vanjapljevaljcic@yahoo.com (V.M.); ljiljanakozic8@gmail.com (L.K.); mandicandjela21@gmail.com (A.D.); marijadrakul@gmail.com (M.D.); dbokonjic@gmail.com (D.B.); miodrag.colic@sanu.ac.rs (M.Č.); 2 Serbian Academy of Sciences and Arts, Kneza Mihajla 35, 11000 Belgrade, Serbia; 3 Medical Faculty of the Military Medical Academy, University of Defense in Belgrade, Crnotravska 17, 11040 Belgrade, Serbia

**Keywords:** dapsone, neutrophils, antioxidant, neutrophil extracellular traps, interleukin 8, phenotype

## Abstract

Dapsone is a sulfone used in treating inflammatory skin conditions. Despite its widespread dermatological use, the pharmacological actions of dapsone remain poorly understood. Here, we examined how different aspects of neutrophil functions are affected by dapsone. Peripheral blood neutrophils from healthy donors were stimulated with phorbol-12-myristate-13-acetate (PMA), N-formyl-L-methionyl-L-leucyl-L-phenylalanine (fMLP), or calcium ionophore (CaI) or primed with cytokines prior to stimulation, in the presence of different concentrations of dapsone (from 10 to 50 µg/mL), followed by analyses of their survival, phenotype, and functional properties. We found that dapsone at the concentration of 50 μg/mL induced a significant neutrophil apoptotic rate during 6 h and 18 h, while other concentrations were well tolerated compared to control non-treated cells. However, dapsone significantly decreased the induced oxidative burst of neutrophils at all non-cytotoxic concentrations. Additionally, dapsone showed a dose-dependent suppression of NETosis in activated neutrophils. The production of IL-8 by dapsone-treated neutrophils was decreased under both stimulated (fMLP) and primed (TNF-α/fMLP) conditions. Moreover, dapsone inhibited the expression of CD11b/CD18, CD66, and CD89 and reversed or significantly mitigated the downregulation of CD16, CD32, CD181, CD88, and CD62L on neutrophils after priming and fMLP stimulation. In conclusion, our results indicate the complexity of dapsone actions on neutrophil functions, extending previous knowledge on the suppression of oxidative burst and IL-8 production upon neutrophils’ activation. Suppressed NETosis and modulation of marker expression associated with different neutrophil functions under inflammatory conditions are new findings, not recognized previously.

## 1. Introduction

Neutrophils are the most abundant immune cell population among blood leukocytes, acting as a critical line of defense against invading pathogens. They are characterized by a remarkable ability to sense the presence of pathogens, followed by their rapid influx from the peripheral circulation to the infected tissue [1,2]. Neutrophil effector functions are mediated by various mechanisms including chemotaxis, phagocytosis, degranulation, production of reactive oxygen species (ROS), release of decondensed chromatin in the form of neutrophil extracellular traps (NETosis), and production of proinflammatory cytokines [3,4,5]. Previously, neutrophils were thought to represent a homogeneous cell population with conserved functions. Recently, a new concept has emerged that presents neutrophils as cells with significant functional diversity [6,7]. Exposure of neutrophils to inflammatory mediators sets them forth in a state of heightened readiness, also known as a primed state. Such neutrophils demonstrate an enhanced capacity to respond more quickly and robustly to additional stimuli [8,9]. For instance, proinflammatory cytokines are commonly used to prime neutrophils for activation by fMLP [10,11]. Furthermore, neutrophils have been recognized as key players in the regulation and coordination of both innate and adaptive immune responses [12,13,14]. Beyond their antimicrobial role, neutrophils play an important part in the inflammatory response to stress or tissue damage in the absence of pathogens. This type of chronic inflammation, known as sterile inflammation, is a hallmark of many autoimmune diseases as well as cancer [15,16]. Many dermatoses are characterized by neutrophil-dominated sterile inflammation [17]. The treatment of choice for these conditions is diaminodiphenyl sulfone, also known as dapsone [18,19].

Dapsone is a sulfone derivative synthesized in the early 20th century [20]. Initially recognized as an antimicrobial agent due to its ability to inhibit folic acid synthesis [21], dapsone has since been predominantly used to treat inflammatory, non-infectious diseases following the discovery of its anti-inflammatory properties. It is indicated for skin conditions such as dermatitis herpetiformis (DH) [22], linear IgA dermatosis [23], erythema elevatum diutinum [24], Sweet syndrome [25], and bullous pemphigoid [26]. Dapsone is also used as an adjuvant therapy for other inflammatory diseases including pyoderma gangrenosum [27], pustular dermatosis [28], leukocytoclastic vasculitis [29], rheumatoid arthritis [30], and cutaneous lupus erythematosus [31]. Despite its widespread use in clinical practice, the pharmacological actions of dapsone remain poorly understood. To date, two primary mechanisms of action have been proposed.The first, the reduction in neutrophil chemotaxis [32,33], and integrin-mediated adherence [34,35] are both considered the dominant modes of action of dapsone. It can also disrupt neutrophil adhesion to IgA and IgG antibodies [36] and downregulate the expression of CD11b, an integrin that plays a crucial role in neutrophil migration, adhesion, and transmigration across blood vessels [37]. Secondly, dapsone has notable antioxidant properties. attributed to its direct scavenging activity and inhibition of ROS production [38,39,40]. This action is thought to prevent further damage to surrounding tissue, the release of pro-inflammatory mediators, and the recruitment of new neutrophils, thus reducing inflammation. Another mechanism of action of dapsone is its ability to modulate the production and secretion of pro-inflammatory mediators, further influencing the inflammatory milieu. This effect is particularly evident in the suppression of cytokines in several human phagocytic and non-phagocytic cell cultures [41,42,43,44].

Despite its known effects, the influence of dapsone on additional aspects of neutrophil function such as NETosis and changes in neutrophil phenotype has not been thoroughly investigated. Therefore, the main objective of this study was to gain a deeper understanding of dapsone’s effects on the function and phenotype of neutrophils under different activation conditions.

## 2. Results

### 2.1. Effect of Dapsone on the Neutrophil Viability

**Figure 1:**
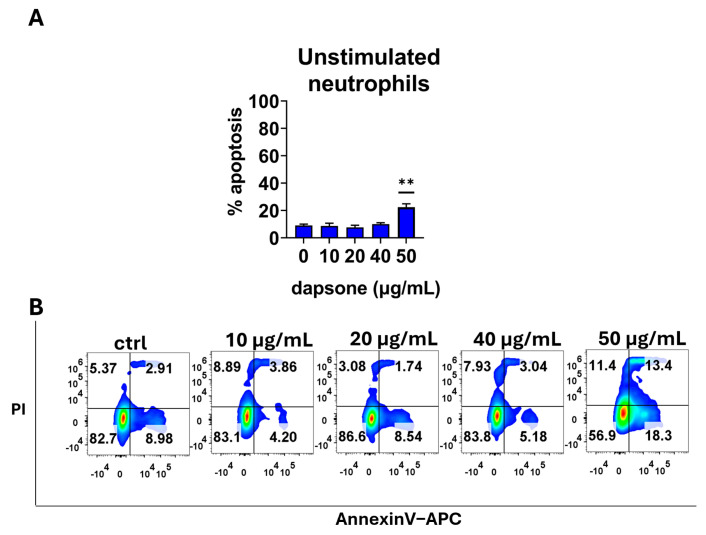
Cytotoxic effect of dapsone on neutrophils. Neutrophils were cultivated alone or with different concentrations of dapsone as indicated for 6 h. Cytotoxicity was evaluated using an Annexin V-APC/Propidium Iodide apoptosis/necrosis assay. (**A**) The summarized results showing % of apoptosis. Values are given as mean ± SD from 3 independent experiments carried out with different donors. ** *p* < 0.01 compared to the corresponding control (dapsone-untreated) group. (**B**) Representative plots of early apoptosis (AnnexineV+), late apoptosis/secondary necrosis (AnnexinV+/Pi+), and primary necrosis (PI+) of neutrophils from one representative experiment. The doublets and the death (FCS low) cells were gated out (see Supplementary Figure S1), and the quadrants were set according to the single-labeled samples.

**Figure 2:**
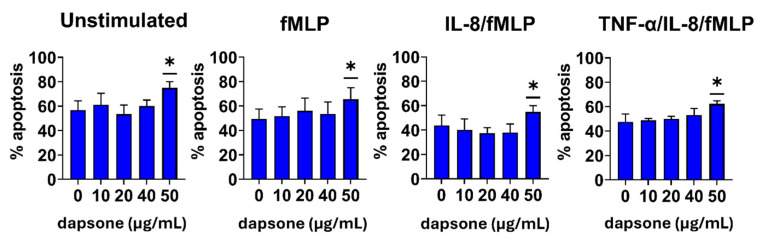
Cytotoxic effect of dapsone on neutrophils. Neutrophils were cultivated alone or with different concentrations of dapsone for 18 h. Cell viability was evaluated by apoptosis/necrosis assay by flow cytometry. Values are given as mean ± SD from 3 independent experiments carried out with different donors. * *p* < 0.05 compared to corresponding control groups (non-treated neutrophils).

Initially, we assessed the effect of dapsone on neutrophil apoptosis, which is a critical function in resolving inflammation. It is known that neutrophils undergo apoptosis at varying rates in peripheral circulation and inflammation sites. Therefore, we measured the apoptotic rate under different conditions (unstimulated or primed/stimulated) and at different time intervals (after 6 h and 18 h) by using apoptosis/necrosis assays. We found that only the highest concentration of dapsone (50 μg/mL) reduced the viability of both resting (unstimulated) and fMLP-activated neutrophils after 6 h (Figure 1) and 18 h (Figure 2) incubation periods due to an increase in the proportion of apoptotic cells (*p* < 0.001 or *p* < 0.05, respectively) compared to corresponding untreated controls. A similar effect was observed when IL-8/fMLP and TNF-α/IL-8/fMLP were used as stimuli. Additionally, the addition of cytokines to fMLP appeared to decrease the spontaneous apoptosis of neutrophils.

### 2.2. Effect of Dapsone on the Production of ROS

**Figure 3:**
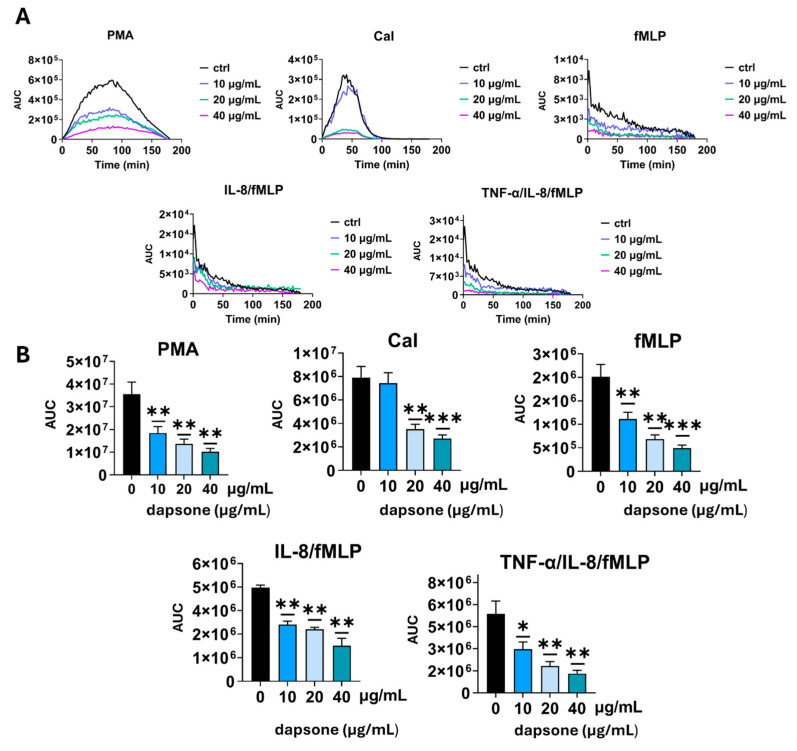
Effect of dapsone on ROS production (**A**,**B**). Neutrophils were cultured alone or with dapsone as described in the Materials and Methods section. ROS production was evaluated by measuring luminescence intensity in real time. The values are shown as the area under the curve (AUC). (**A**) A representative histogram showing real-time ROS production. (**B**) Summarized results are presented as mean ± SD from 4 independent experiments carried out with different donors. * *p* < 0.05; ** *p* < 0.01 and *** *p* < 0.001 compared with corresponding control (dapsone-untreated) groups.

After evaluating dapsone’s impact on viability, we examined its potential to alter the production of ROS by neutrophils by measuring luminescence in real-time. Neutrophils were treated with dapsone in doses of 10, 20, and 40 µg/mL. As shown (Figure 3), all three dapsone concentrations showed a significant dose-dependent capacity to reduce the production of ROS when neutrophils were stimulated with PMA or fMLP, in contrast to CaI, where the lowest concentration was non-modulatory. IL-8/TNF-α-priming significantly enhanced the production of ROS, while the cells primed with IL-8 alone were similar to non-primed cells in their capacity to produce ROS upon fMLP stimulation. The three highest dapsone concentrations showed a significant dose-dependent capacity to reduce the production of (ROS) in both PMA- and all fMLP-stimulated groups, while only the two highest concentrations displayed a dose-dependent decrease under CaI stimulation. In the unstimulated group, there was no difference compared to control.

### 2.3. Effect of Dapsone on NETosis

**Figure 4:**
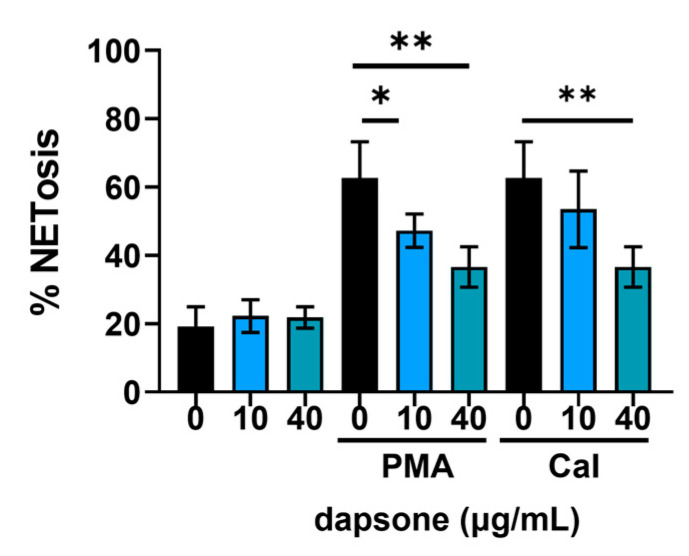
Effect of dapsone on NETosis. Neutrophils were treated with two concentrations of dapsone and stimulated for 4 h. Afterward, the cells were stained with the fluorescent dye Syto Green to measure NETosis by fluorescence intensity. The results were presented as a percentage of NETosis. Values are given as mean ± SD from 3 independent experiments carried out with different donors. * *p* < 0.05 and ** *p* < 0.01 compared to the corresponding control groups, as indicated.

One of the most relevant aspects of neutrophil function is the formation of neutrophil extracellular traps (NETs) [3]. The degree of NETosis was measured as the intensity of DNA fluorescence after 4 h of incubation, relative to total DNA (100%). NETosis was induced by PMA or CaI following treatment with dapsone (10 μg/mL and 40 μg/mL). Resting neutrophils were also assessed. In the PMA-stimulated group (Figure 4), dapsone showed a dose-dependent suppression of NETosis. In contrast, in the CaI-stimulated group, only the higher dose led to a significant decrease. In the resting group, there was no difference compared to the control.

### 2.4. Effect of Dapsone on the Production of IL-8 by Neutrophils

**Figure 5:**
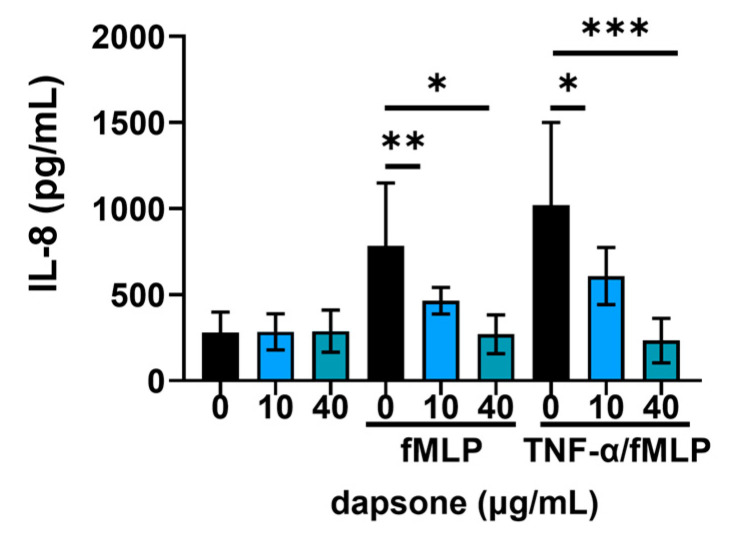
Effect of dapsone on the secretion of IL8. After incubation with two concentrations of dapsone (10 μg/mL and 40 μg/mL), neutrophils were either left resting or stimulated with fMLP or primed with TNF-α before stimulation. The levels of IL-8 in culture supernatants were measured by a commercial ELISA kit. The summarized results are presented as mean ± SD from 5 independent experiments carried out with different donors. * *p* < 0.05, ** *p* < 0.01, and *** *p*< 0.001 between the indicated samples.

Then, we assessed the levels of IL-8 from the supernatants after 18 h of incubation. Priming with TNFα appeared to be the strongest inducer of IL-8 secretion in all the groups (Figure 5). We found that dapsone significantly reduced the production of IL-8 in a dose-dependent manner in both stimulated cultures. However, no significant difference in IL-8 production was observed in resting neutrophils.

### 2.5. Effect of Dapsone on the Neutrophil Phenotype

The impact of dapsone on neutrophil phenotype was analyzed by using flow cytometry. After incubation with dapsone (10 μg/mL and 40 μg/mL), neutrophils were primed with cytokine cocktails (as in the viability assay) and subsequently stimulated with fMLP, following the procedure detailed in the Materials and Methods.

**Figure 6:**
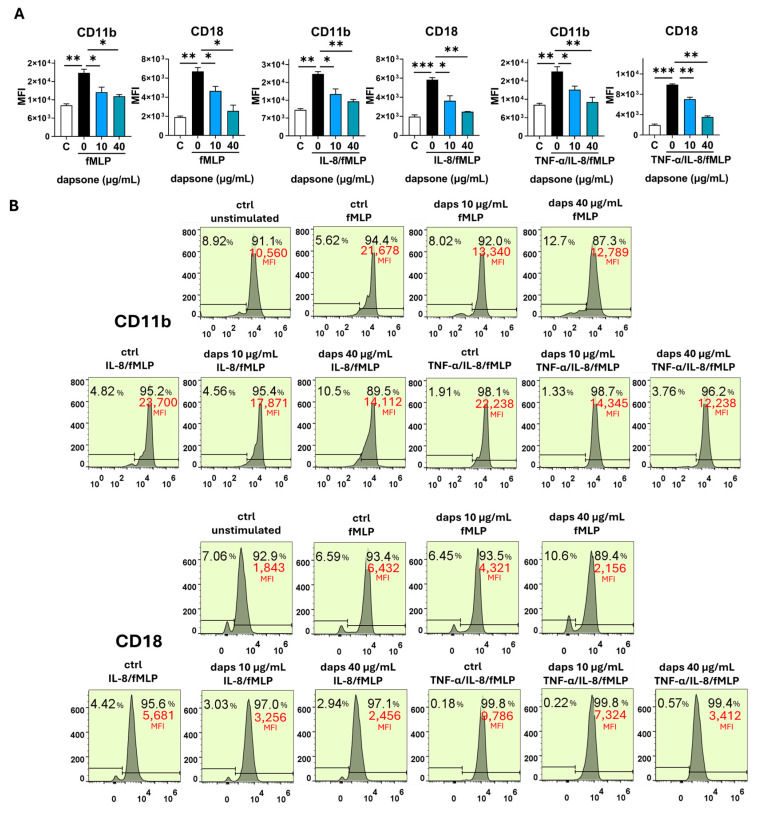
Effect of dapsone on the neutrophil phenotype. After incubation with dapsone (10 μg/mL and 40 μg/mL) neutrophils were activated with fMLP or primed with cytokines and then subsequently stimulated with fMLP. (**A**) The expressions of CD11b and CD18 are represented as mean fluorescence intensity (MFI) (mean ± SD from 3 independent experiments). (**B**) The representative histograms from one experiment. * *p* < 0.05, ** *p* < 0.01, and *** *p* < 0.001 compared with corresponding controls as indicated. C = control, unstimulated neutrophils.

**Figure 7:**
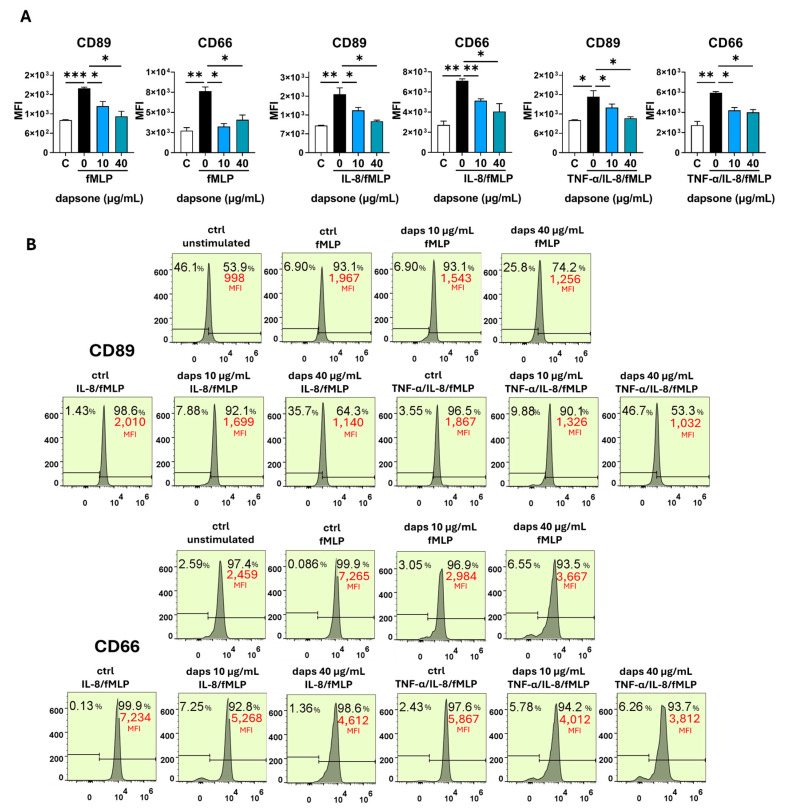
Effect of dapsone on the phenotype of primed neutrophils. After incubation with dapsone (10 μg/mL and 40 μg/mL), neutrophils were activated with fMLP or primed with cytokines and then subsequently stimulated with fMLP. (**A**) The expressions of CD89 and CD66 are represented as mean fluorescence intensity (MFI) (mean ± SD from 3 independent experiments). (**B**) The representative histograms from one experiment. * *p* < 0.05, ** *p* < 0.01, and *** *p* < 0.001 compared with corresponding controls as indicated. C = control, unstimulated neutrophils.

**Figure 8:**
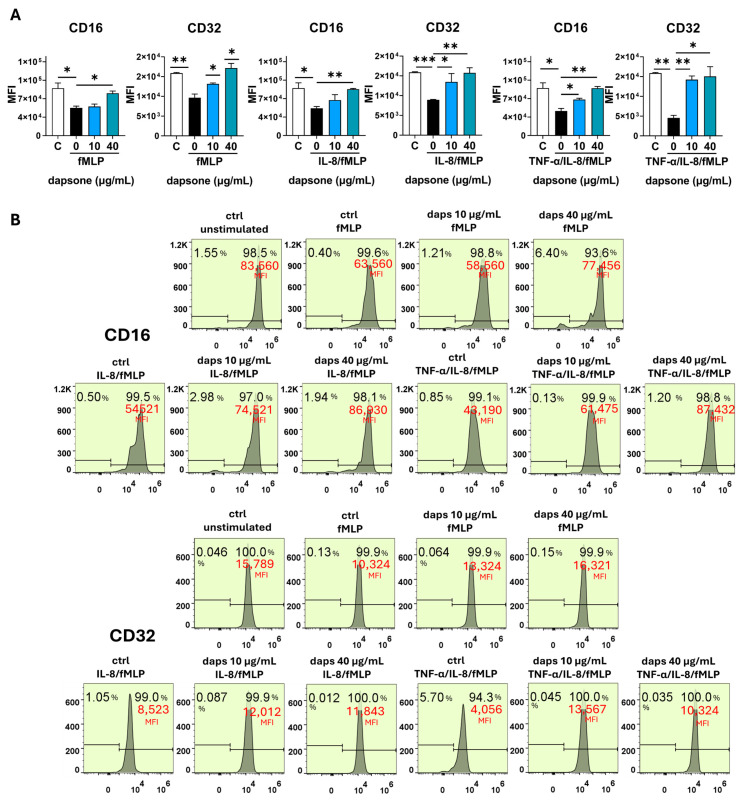
Effect of dapsone on the neutrophil phenotype. After incubation with dapsone (10 μg/mL and 40 μg/mL), neutrophils were activated with fMLP or primed with cytokines and then subsequently stimulated with fMLP. (**A**) The expressions of CD16 and CD32 are represented as mean fluorescence intensity (MFI) (mean ± SD from 3 independent experiments). (**B**) The representative histograms from one experiment. * *p* < 0.05, ** *p* < 0.01, and *** *p* < 0.001 compared with corresponding controls as indicated. C = control, unstimulated neutrophils.

**Figure 9:**
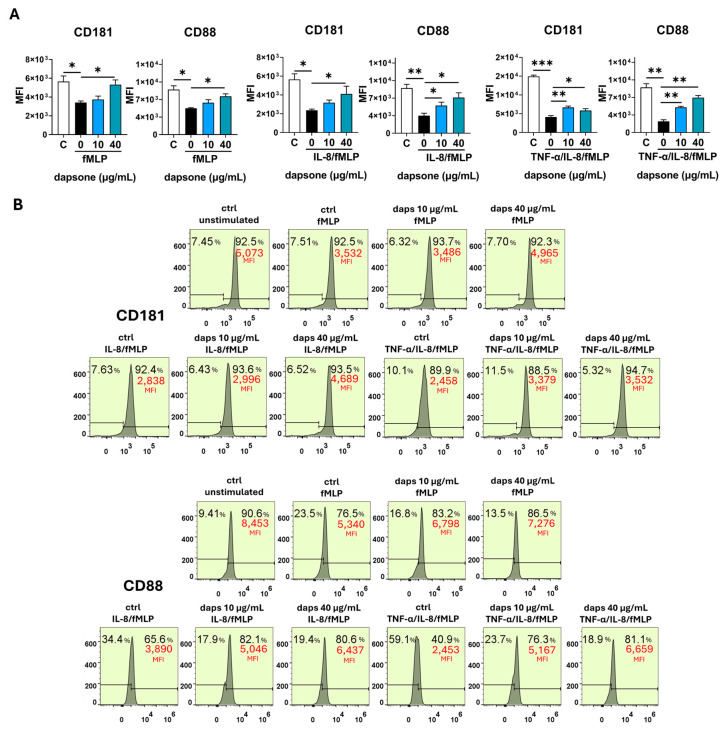
Effect of dapsone on the neutrophil phenotype. After incubation with dapsone (10 μg/mL and 40 μg/mL), neutrophils were activated with fMLP or primed with cytokines and then subsequently stimulated with fMLP. (**A**) The expressions of CD181 and CD88 are represented as mean fluorescence intensity (MFI) (mean ± SD from 3 independent experiments). (**B**) The representative histograms from one experiment. * *p* < 0.05, ** *p* < 0.01, and *** *p* < 0.001 compared with corresponding controls as indicated. C = control, unstimulated neutrophils.

**Figure 10:**
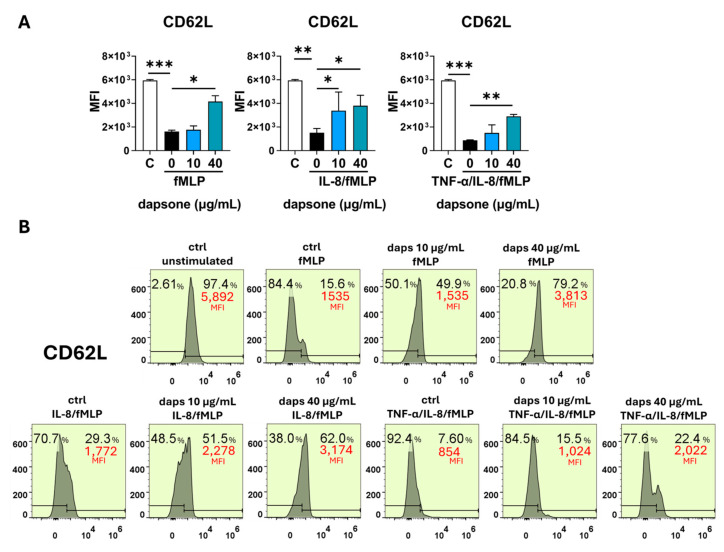
Effect of dapsone on the neutrophil phenotype. After incubation with dapsone (10 μg/mL and 40 μg/mL), neutrophils were activated with fMLP or primed with cytokines and then subsequently stimulated with fMLP. (**A**) The expression of CD62L is represented as mean fluorescence intensity (MFI) (mean ± SD from 3 independent experiments). (**B**) The representative histograms from one experiment. * *p* < 0.05, ** *p* < 0.01, and *** *p* < 0.001 compared with corresponding controls as indicated. C = control, unstimulated neutrophils.

Stimulation with fMLP significantly increased the expression of CD11b, CD18, CD89, and CD66, while markedly decreasing CD16, CD62L, CD32, CD181, and CD88 expression. These effects were further amplified by cytokine priming, especially with IL-8/TNF-α (Figure 6, Figure 7, Figure 8, Figure 9 and Figure 10). Dapsone treatment significantly influenced the expression of all analyzed markers in primed and stimulated neutrophils but had no effect on resting neutrophils. The higher dapsone concentration (40 μg/mL) notably altered marker expression, as reflected by changes in mean fluorescence intensity (MFI) across all treated groups (Figure 6, Figure 7, Figure 8, Figure 9 and Figure 10). Specifically, dapsone almost completely reversed (CD32 and CD16) or significantly mitigated the downregulation of CD62L, CD181, and CD88, while inhibiting the upregulation of CD11b, CD18, CD89, and CD66.

In contrast, the lower dapsone concentration (10 μg/mL) showed somewhat different modulatory effects. It influenced CD89, CD32, and CD66 expression patterns similarly to the higher concentration across all groups but modulated CD88 expression only after cytokine priming. CD11b/CD18 expression was significantly suppressed in the fMLP and TNF-α/IL-8 groups, whereas CD16 and CD181 expression were altered primarily after IL-8/TNF-α priming. CD62L expression was specifically modulated following IL-8 priming.

Comparable trends were observed in the analysis of the percentage of positive cells, except for markers with consistently high expression levels (80–99%), such as CD16, CD81, CD66, CD32, CD11b, and CD18 (Supplementary Figures S2–S4).

## 3. Discussion

Due to its anti-inflammatory and immunosuppressive properties, dapsone has been used as a therapy of choice for the treatment of autoimmune and autoinflammatory skin conditions, characterized by excessive neutrophil infiltration [45]. Dapsone has also been introduced as an adjuvant therapy for many other dermatoses with different etiologies, often in combination with other topical or systemic anti-inflammatory drugs [46]. Its great clinical efficacy has been attributed to its antioxidative properties, as well as its ability to inhibit neutrophil recruitment to affected skin tissue [32,33,38,39,40]. However, the clinical effectiveness of dapsone cannot be fully explained by this mechanism, alone. Therefore, the main objective of this study was to gain a deeper understanding of dapsone’s effects on the function and phenotype of neutrophils under different activation conditions.

Dapsone is most frequently used as the first-line treatment for skin conditions primarily associated with the deposition of autoantibodies in skin tissue (such as DH and IgA linear dermatosis) [47,48]. The specific antibody–antigen interaction initiates a series of immunological reactions including activation of tissue-resident immune cells and keratinocytes, increased production of proinflammatory cytokines, and activation of the complement system [49,50,51,52,53]. This inflammatory cascade leads to the recruitment of neutrophils from the periphery, activating their effector mechanisms, which further elevates the inflammatory response and causes tissue damage, characterized by skin lesions [54].

First, we investigated the cytotoxic effects of dapsone on neutrophils to determine appropriate concentrations for subsequent experiments. We found that neutrophils tolerated dapsone well up to 40 µg/mL but exhibited significant toxicity at 50 µg/mL. These results are consistent with findings from Kwon et al., who observed similar cytotoxic effects in mouse bone marrow cell lines [43]. The lower basal apoptosis of neutrophils when IL-8 or TNF-α are used as stimuli, together with fMLP, can be explained by the anti-apoptotic effects of these cytokines [55,56]. Other authors have reported in vitro pharmacological effects of dapsone at concentrations ranging from 20 µg/L up to 300 µg/mL, depending on the functions examined and cell lines used [35,57]. In vivo, maximum plasma concentrations after single oral doses of dapsone (50–300 mg) were in the range of 0.63 to 4.82 mg/L [19]. Although these concentrations are lower, therapeutic effects are usually achieved with prolonged treatment.

In the following segment, we examined how dapsone treatment affects neutrophil effector functions. We utilized stimuli such as PMA and CaI to mimic physiological activation of the protein kinase C (PKC) [58] pathway and calcium signaling [59,60], both of which are essential for neutrophil activation and function. Additionally, we used fMLP to simulate neutrophil activation through chemokine receptor signaling via G protein-coupled receptors and the phospholipase C/diacylglycerol (PLC/DAG) pathway [61,62].

The most significant alterations in neutrophil functions were observed in ROS production and IL-8 levels. Suda et al. demonstrated that dapsone impaired the production of superoxide anion (O_2_^−^) only in fMLP-stimulated neutrophils, by directly interfering with calcium influx, with no changes observed in PMA-stimulated cultures even at higher doses [40]. However, our findings differ from those results. We showed a notable reduction in ROS production across groups stimulated with fMLP (alone or in combination with cytokines such as TNF-α and IL-8), PMA, or CaI. These differences could be attributed to differences in the types of oxygen radicals measured or to methodological variations. Additionally, it has been suggested that dapsone exhibits antioxidant properties by directly neutralizing oxygen radicals [37,38,39]. Considering that, we could assume that dapsone exerts antioxidative effects both by interfering with signal transduction pathways and through direct radical scavenging, particularly in response to PMA stimulation.

IL-8 plays a central role in the pathology of neutrophil dermatoses. The elevated levels detected in both serum and skin tissue of DH patients are believed to drive significant neutrophil infiltration [51,52,63]. Our data demonstrate dapsone’s inhibitory effect on IL-8 production, supporting previous findings from studies using models of human bronchial epithelial cells [41], peripheral blood mononuclear cells [42], and human keratinocytes [44]. Thus, reducing IL-8 production may be one of the mechanisms by which dapsone exhibits its beneficial clinical effect.

For the first time, we evaluated the effect of dapsone on the release of NET. We observed that dapsone impairs NET formation following stimulation with both PMA and CAI. This effect may be linked to dapsone’s antioxidant properties, particularly its radical scavenging potential [64]. Notably, the prominent presence of NETs has been reported in the skin lesions of certain neutrophilic dermatoses treated with dapsone, including Sweet syndrome, pyoderma gangrenosum, and subcorneal pustular dermatosis, suggesting a potential role in their development [65]. Neutrophils exhibit high plasticity and the ability to adapt their effector function to the environment. These adaptations are manifested through the variable expression of surface markers, that are associated with different effector functions [66,67]. The expression of CD11b/CD18 (β2 integrin), CD66, and CD89 was upregulated on both activated (fMLP-stimulated) and cytokine (IL-8 or IL-8/TNF-α)-primed/activated neutrophils, and these results are consistent with previous findings [68,69,70]. All these markers were significantly downregulated by dapsone dose-dependently. The downregulatory effects of dapsone on these markers align with its proposed beneficial role in inhibiting chemotaxis, transmigration, degranulation, ROS production, and other neutrophil functions associated with inflammation.

CD11b/CD18 plays a crucial role in neutrophil transmigration and adhesion to the extracellular matrix, processes essential for neutrophil activation [37]. Our results support earlier studies demonstrating that dapsone reduces CD11b expression and inhibits CD11b/CD18-mediated neutrophil adhesion [34,35]. Additionally, neutrophils from patients with DH undergoing dapsone treatment exhibit reduced chemotaxis compared to those from untreated patients. The rapid therapeutic response observed in these patients further suggests that inhibition of neutrophil migration may be a key mechanism underlying the pharmacological effects of dapsone [71].

CD89, also known as FcαRI, binds antigen–IgA complexes and triggers various immunological responses, including respiratory burst and degranulation [72]. The inhibitory effect of dapsone on CD89 expression aligns with published data showing that dapsone reduces neutrophils’ capacity to bind IgA antibodies in vitro via CD89 [73]. However, these in vitro findings do not correspond to observations in DH patients undergoing dapsone therapy. In these patients, neutrophil CD89 exhibited an increased capacity to bind IgA antibodies, which remained unchanged after dapsone treatment [74].

CD66, primarily recognized as a marker of specific granule degranulation, also plays a key role in neutrophil β2 integrin-mediated functions such as transmigration, adhesion to the extracellular matrix, and the induction of respiratory burst. Furthermore, CD66 facilitates the secretion of stored IL-8 [75]. In this context, the downregulatory effect of dapsone on CD66 expression observed in our study represents an additional mechanism contributing to its anti-inflammatory properties.

Several neutrophil markers (CD62L, CD16, CD181, CD32, and CD89) were significantly downregulated following fMLP activation, an effect that was further amplified when neutrophils were primed with pro-inflammatory cytokines, particularly the IL-8/TNF-α cocktail. Dapsone effectively reversed these suppressive effects, either almost completely (for CD32 and CD16) or partially (for CD62L, CD181, and CD89). Notably, the restorative effect of dapsone was generally more pronounced at higher concentrations compared to lower ones.

CD32, or FcγRII, is a low-affinity receptor for monomeric IgG that promotes respiratory burst and neutrophil degranulation. CD16, also referred to as FcγRIII, is another low-affinity receptor for monomeric IgG, contributing to neutrophil degranulation and respiratory burst in response to immune complexes [76]. However, in this context, CD16 is less potent than CD32 in enhancing neutrophil functions [77].

The downregulation of CD16 on neutrophils upon activation is attributed to its shedding, mediated by the ADAM17 metalloprotease [78]. This mechanism may account for our findings. While CD16 downregulation has been shown to correlate with increased neutrophil apoptosis [79], this is likely not relevant to our study, as activated neutrophil apoptosis was lower compared to unstimulated cells. The mechanisms underlying CD32 downregulation remain unclear, particularly given that the release of CD16 can enhance signaling through CD32 [78].

CD181, also known as CXCR1, is a receptor for IL-8. Binding of IL-8 to CD181 induces neutrophil chemotaxis while also triggering degranulation and respiratory burst [80,81]. Similarly, CD88, the C5a receptor, directs neutrophils to sites of inflammation by responding to the complement protein C5a, which is produced during complement activation [82]. This receptor plays a critical role in initiating degranulation, respiratory burst, and chemotaxis [83]. Both fMLP and IL-8 have been shown to downregulate the expression of CD181 and CD88, likely as a mechanism to terminate or reduce signal transduction [80]. Additionally, the shedding of CD62L on activated neutrophils is a well-documented phenomenon [6] and is consistent with our findings. CD62L, or L-selectin, belongs to the family of surface lectins and plays a key role in the “rolling” of neutrophils along the endothelium, a critical step in the initiation of the inflammatory process. Moreover, CD62L supports neutrophil transmigration by stabilizing interactions between CD11b/CD18 and the endothelium [84]. Although our results demonstrate the modulatory role of dapsone on CD62L expression, no change in the expression of this marker was reported in patients undergoing dapsone therapy.

An important question relates to the significance of the modulatory effect of dapsone on the expression of CD16, CD32, CD88, CD181, and CD62L. A general explanation can be viewed through the hypothesis that reduced expression of these molecules on activated neutrophils may promote inflammation in various ways. If this assumption is correct, dapsone’s ability to reverse or mitigate these effects could be considered a potentially beneficial anti-inflammatory mechanism. However, further research is needed to fully understand its implications.

In conclusion, our findings indicate that dapsone exerts a complex anti-inflammatory effect, primarily through its antioxidative mechanism and by reducing overall neutrophil activation, with the greatest impact observed on primed neutrophils. To fully elucidate the role of dapsone, further investigation is needed to explore its effects on neutrophils stimulated with other physiological stimuli. Additionally, clarifying the precise molecular mechanisms of dapsone remains a priority for future research.

## 4. Materials and Methods

### 4.1. Isolation of Neutrophils

Peripheral blood was sampled by venipuncture from healthy adult volunteers using K_2_EDTA collection tubes. Neutrophils were isolated according to a protocol that combines dextran and density gradient centrifugation Briefly, after the sedimentation of the sampled blood with 3% dextran solution (Sigma-Aldrich, Steinheim, Germany), to remove erythrocytes, leukocyte-enriched plasma was layered on a Lymphoprep gradient medium (density, 1.077 g/mL) (PAA Laboratories, Vienna, Austria) to separate mononuclear cells. After centrifugation, the granulocyte-rich layer was collected, and residual erythrocytes were lysed for 2 min at room temperature using a lysing buffer (NH_4_Cl + KHCO_3_ + Na_2_EDTA). Granulocytes were washed once and resuspended in Hanks’ balanced salt solution (HBSS) medium (Sigma-Aldrich) without Ca^2+^ and Mg^2+^. Cell count and viability were determined by staining the cell suspension with 0.4% Trypan blue solution (Sigma-Aldrich).

### 4.2. Apoptosis Assay

Apoptosis of neutrophils induced by dapsone was evaluated using a commercial apoptosis detection kit (Biolegend, London, UK) containing Annexin V conjugate with allophycocyanin (APC) and propidium iodide (PI). Neutrophils were incubated in polypropylene 2 mL tubes (Eppendorf, Hamburg, Germany) at a density of 3 × 10^5^/200 µL per tube in HBSS medium with Ca^2+^ and Mg^2+^ (Sigma-Aldrich), 1% heat-inactivated human serum, and 10 mM HEPES (Sigma-Aldrich). This was followed by treatment with dapsone (50 μg/mL 40 μg/mL, 20 μg/mL, 10 μg/mL, or 0 μg/mL) (Sigma-Aldrich) in PBS, for 1 h at 37 °C and 5% CO_2_. To evaluate whether the activation status of neutrophils influences their susceptibility to apoptosis induced by dapsone, we used both resting neutrophils and neutrophils activated with different combinations of stimuli, such as fMLP (1 µM), or fMLP (1 µM) in combination with priming with IL-8 (20 ng/mL) or both IL-8 (20 ng/mL) and TNF-α (20 ng/mL). Because the levels of these cytokines are known to be elevated in the serum and tissues of patients suffering from DH, we chose them to mimic the environment to which dapsone-treated neutrophils would be exposed [51,63]. Cytokines were purchased from Biolegends, UK, and fMLP was purchased from Sigma-Aldrich. Following cell priming (30 min, at 37 °C), neutrophils were stimulated with fMLP for either 6 h or 18 h. After incubation, neutrophilswere stained with Annexin V-APC/PI for 20 min in the dark at room temperature according to the manufacturer’s instructions. The samples were analyzed using flow cytometry (Attune, Thermo Fisher Scientific, Waltham, MA, USA).

### 4.3. The Production of ROS

A chemiluminescence assay with luminol was used to measure the kinetics of intra- and extracellular ROS production. Neutrophils in HBSS medium with Ca^2+^ and Mg^2+^, 1% serum, and 10 Mm HEPES were seeded in a 96-well white flat-bottomed plate (Corning, NY, USA at a density of 2.5 × 10^5^/100 µL per well and treated with three non-toxic dapsone concentrations (40 μg/mL, 20 μg/mL, or 10 μg/mL) for 1 h at 37 °C and 5% CO_2_. Subsequently, 50 μL luminol (Serva, Munich, Germany) was added to each well. After a 15-min incubation, the cells were stimulated with a single stimulus: phorbol myristate acetate (PMA, Sigma Aldrich) (20 nM), calcium ionophore A23187 (CaI, Sigma Aldrich), or fMLP (1 µM). Alternatively, they were stimulated with different combinations of priming agents: IL-8 (20 ng/mL) alone, or IL-8 (20 ng/mL) together with TNF-α (20 ng/mL), followed by stimulation with fMLP (1 µM). The chemiluminescence resulting from ROS release was analyzed immediately after stimulation with a chemiluminescent spectrometer (Synergy HTX, Bio Tek Instruments, Santa Clara, CA, USA). The generation of ROS was monitored in real-time, every two minutes for a period of 3 h at 37 °C and 5% CO_2_. The intensity of emitted light was directly proportional to the production of ROS.

### 4.4. NETosis

Free-DNA staining with a cell-impermeable fluorescent dye (Sytox green) (Invitrogen/Thermo Fisher, Carlsband, CA, USA) was used to measure the NETosis. Neutrophils in HBSS medium with Ca^2+^ and Mg^2+^ enriched with 1% heat-inactivated human serum and 10 mM HEPES were seeded in the 96-well white flat-bottomed plate (Corning, NY, USA) at a density of 2.5 × 10^5^/100 µL per well and treated with dapsone for 1 h at 37 °C and 5% CO_2_. For the following experiments, we chose two concentrations that exhibited different effects in the previous experiment (40 μg/mL and 10 μg/mL). After incubation, neutrophils were left unstimulated or stimulated with two NETosis inducers: PMA (50 nM) or CaI (1 µM). Triton X-100 (1%) (Sigma-Aldrich) was added in corresponding control wells for cell permeabilization and to measure total DNA release in the cultures (corresponding to 100% of free DNA). Cells were incubated for 4 h at 37 °C. Subsequently, a fluorescent dye, Sytox green, was added to each well at a final concentration of 50 nM and incubated for 15 min at 37 °C. The fluorescence intensity was analyzed using a fluorimeter (Synergy HTX, Bio Tek Instruments, Santa Clara, CA, USA). The fluorescence intensity was directly proportional to the concentration of free DNA.

### 4.5. Determination of IL-8 in Culture Supernatants

The secretion of IL-8 was measured from unstimulated and stimulated culture supernatants using a commercial sandwich ELISA kit (R&D Systems, Minneapolis, MN, USA). Following treatment with two dapsone concentrations (40 μg/mL and 10 μg/mL), neutrophils were stimulated with fMLP (1 µM) or fMLP (1 µM) + TNF-α (20 ng/mL) for 18 h at 37 °C. After incubation, the supernatants were collected and analyzed according to the manufacturer’s instructions.

### 4.6. Phenotypic Analysis of Neutrophils with Flow Cytometry

The analysis of neutrophils’ phenotypical characteristics was determined by using the panel of fluorescent-labeled monoclonal antibodies against the following surface markers: we assessed the expression of several surface markers known as markers of neutrophil activation: CD16-FITC (Fc fragment of IgG receptor, FcγRIII; 1:200), CD62L-AlexaFlour488 (L-selectin; 1:100), CD181-FITC (high-affinity IL8 receptor, CXCR1; 1:100), CD88-FITC (complement component 5a receptor, C5AR1; 1:100), CD32-PECy7 (Fc fragment of IgG receptor FcγRII receptor; 1:100), CD89-PecCP (Fc fragment of IgA receptor, FcαR receptor; 1:100), CD66-APC (marker of degranulation specific granules; 1:100), and CD11b-PE/CD18-AlexaFlour700 (β2 integrin, 1:100). The antibodies were obtained from Biolegend (San Diego, CA, USA). Neutrophil granulocytes were cultivated in polypropylene 2 mL tubes at a density of 5 × 10^5^/300 µL per tube in HBSS medium with Ca^2+^ and Mg^2+^ supplemented with 1% heat-inactivated human serum and 10 mM HEPES following the incubation with dapsone (40 μg/mL and 10 μg/mL) for 1h. Control cells were cultivated in medium only. After incubation, neutrophils were stimulated with fMLP (1 µM). Before stimulation, a portion of the culture was primed with cytokines for 30 min at 37 °C, as previously described, and then stimulated with fMLP for 1 h. After cultivation, neutrophils were collected, washed, and resuspended in cold phosphate-buffered saline (PBS) supplemented with 0.01% sodium azide (NaN3) and 2% FCS. Blocking antibodies for Fc receptors were added (1:50, 25 min, 4 °C), followed by the addition of monoclonal antibodies (30 min, 4 °C). Afterward, cells were assessed with a flow cytofluorimeter (Attune, Thermo Fisher Scientific). The results were presented as the percentage of cells positive for these markers and mean fluorescence intensity (MFI) as a measure of expression density for each marker. Doublets and dead cells were excluded by gaiting neutrophils according to their specific side scatter (SSC)/forward scatter (FSC) properties. Before each experiment, the signal overlap between the fluorescence channels was compensated for by using single-stain controls. The analysis was conducted with FlowJo V10software.

### 4.7. Statistical Analysis

Results are shown as either representative examples or as the mean ± standard deviation (SD) from at least three independent experiments. Within each experiment, samples were tested in triplicate. Differences between the treatments were analyzed by repeated measures ANOVA with Dunnett’s multiple comparison test or a *t* test (paired or unpaired). *p*-values of 0.05 or less were considered statistically significant. All analyses were performed using GraphPad Prism 9 software (GraphPad, La Jolla, CA, USA).

## 5. Conclusions

Our findings highlight the complexity of dapsone’s effects on neutrophil functions, expanding the existing knowledge on its ability to suppress oxidative bursts during neutrophil activation. Notably, we observed a reduction in IL-8 production by dapsone-treated neutrophils under both stimulated (fMLP) and primed (TNF-α/fMLP) conditions. For the first time, dapsone has been shown to suppress NETosis. Furthermore, the modulation of key markers critical to neutrophil function in response to cytokine priming and fMLP stimulation suggests anti-adhesive, anti-chemotactic, anti-oxidative, and other anti-inflammatory effects of dapsone on neutrophils. These results pave the way for further investigations into the molecular mechanisms underlying dapsone’s actions.

## Data Availability

The data presented in this study are available in this article.

## Supplementary Materials

The following supporting information can be downloaded at https://www.mdpi.com/article/10.3390/molecules30010113/s1: Figure S1: The strategy for gating the neutrophil population in the analysis of their phenotype following dapsone treatment and stimulation. The plots, from one representative experiment out of three with similar results, show the percentages (%) of singlet cells, total neutrophils, and live (Propidium Iodide—PI negative) neutrophils. Figure S2: Effect of dapsone on the neutrophil phenotype. After incubation with dapsone (10 μg/mL and 40 μg/mL), neutrophils were exposed to fMLP. The summarized results of surface marker expression (CD16, CD62L, CD181, CD88, CD89, CD66, CD32, CD11b, and CD18) are presented as the percentage (%) of marker-expressing cells ± SD from 3 independent experiments. * *p* < 0.05, ** *p* < 0.01, and *** *p* < 0.001, compared with the corresponding controls, as indicated. C = control, unstimulated neutrophils. Figure S3: Effect of dapsone on the neutrophil phenotype. After being exposed to dapsone (10 μg/mL and 40 μg/mL) neutrophils were primed with IL-8 and then stimulated with fMLP. The summarized results of surface marker expression (CD16, CD62L, CD181, CD88, CD89, CD66, CD32, CD11b, and CD18) are presented as the percentage (%) of marker-expressing cells ± SD from 3 independent experiments. * *p* < 0.05, ** *p* < 0.01, and *** *p* < 0.001, compared with the corresponding controls, as indicated. C = control, unstimulated neutrophils. Figure S4: Effect of dapsone on the neutrophil phenotype. After being treated with two concentrations of dapsone (10 μg/mL and 40 μg/mL) neutrophils were primed with TNF-α and IL-8, and subsequently stimulated with fMLP. The summarized results of surface marker expression (CD16, CD62L, CD181, CD88, CD89, CD66, CD32, CD11b, and CD18) are presented as the percentage (%) of marker-expressing cells ± SD from 3 independent experiments. * *p* < 0.05, ** *p* < 0.01, and *** *p* < 0.001, compared with the corresponding controls, as indicated. C = control, unstimulated neutrophils.
