# Supplementary material for: Dapsone Alters Phenotypical and Functional Properties of Human Neutrophils In Vitro
Source: Molecules. 2024 Dec 30;30(1):113. doi: 10.3390/molecules30010113 (PMC11722540; doi:10.3390/molecules30010113)
Supplement: Supplementary file 1 [file molecules-30-00113-s001.zip › molecules-3265715-supplementary.pdf]

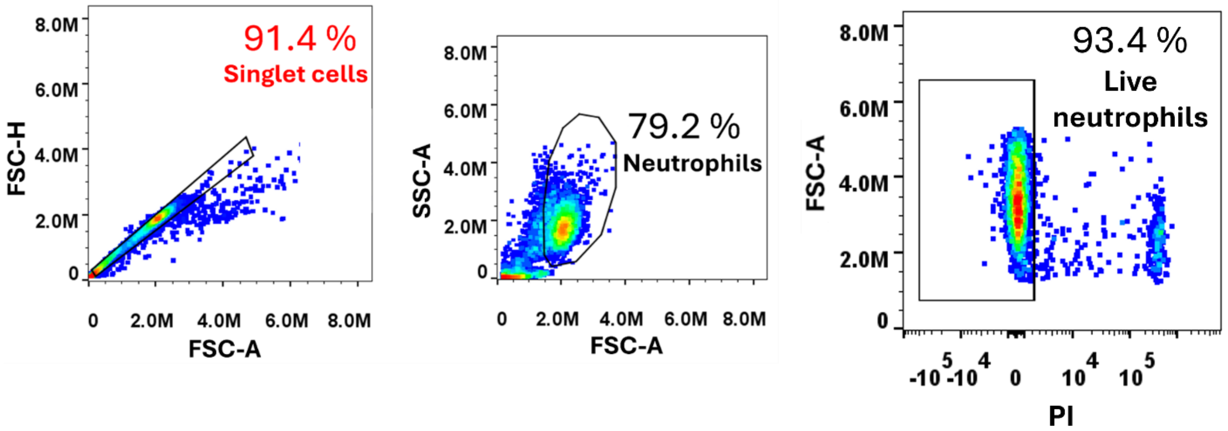

Supplementary Figure S1. The strategy for gating the neutrophil population in the analysis of their phenotype following dapsone treatment and stimulation. The plots, from one representative experiment out of three with similar results, show the percentages (%) of singlet cells, total neutrophils, and live (Propidium Iodide - PI negative) neutrophils.

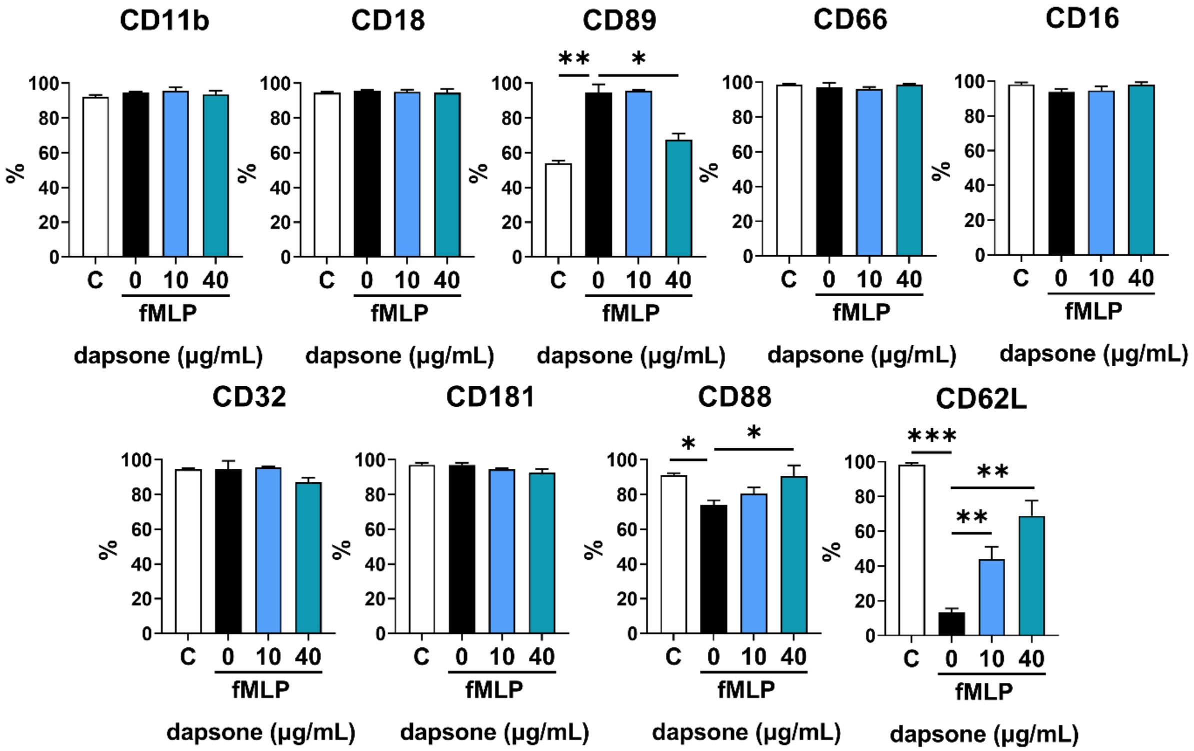

Supplementary Figure S2. Effect of dapsone on the neutrophil phenotype. After incubation with dapsone (10 µg/mL and 40 µg/mL), neutrophils were exposed to fMLP. The summarized results of surface marker expression (CD16, CD62L, CD181, CD88, CD89, CD66, CD32, CD11b, and CD18) are presented as the percentage (%) of marker-expressing cells ± SD from 3 independent experiments. *p < 0.05, **p < 0.01, and *p < 0.001, compared with the corresponding controls, as indicated. C = control, unstimulated neutrophils

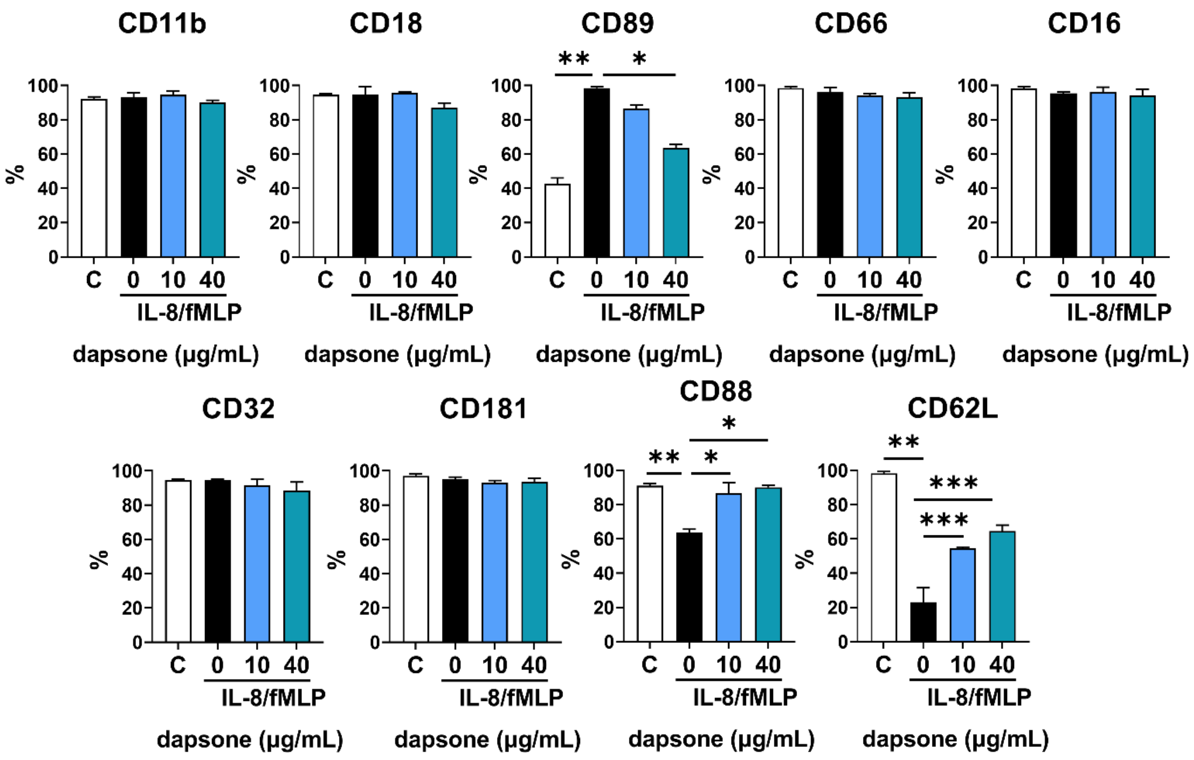

Supplementary Figure S3. Effect of dapsone on the neutrophil phenotype. After being exposed to dapsone (10 μg/mL and 40 μg/mL) neutrophils were primed with IL-8 and then stimulated with fMLP. The summarized results of surface marker expression (CD16, CD62L, CD181, CD88, CD89, CD66, CD32, CD11b, and CD18) are presented as the percentage (%) of marker-expressing cells ± SD from 3 independent experiments. *p < 0.05, **p < 0.01, and *p < 0.001, compared with the corresponding controls, as indicated. C = control, unstimulated neutrophils.

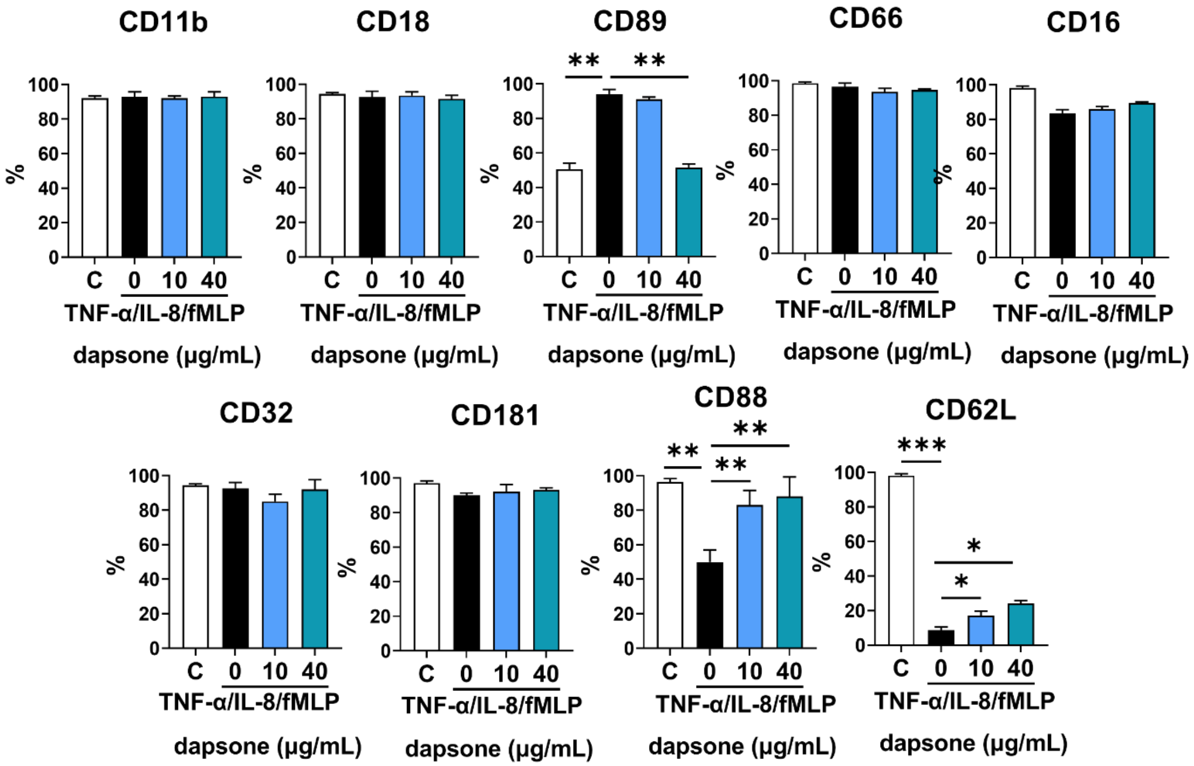

Supplementary Figure S4. Effect of dapsone on the neutrophil phenotype. After being treated with two concentrations of dapsone (10 µg/mL and 40 µg/mL) neutrophils were primed with TNF-*α* and IL-8, and subsequently stimulated with fMLP. The summarized results of surface marker expression (CD16, CD62L, CD181, CD88, CD89, CD66, CD32, CD11b, and CD18) are presented as the percentage (%) of marker-expressing cells ± SD from 3 independent experiments. *$p < 0.05$, **$p < 0.01$, and *$p < 0.001$, compared with the corresponding controls, as indicated. C = control, unstimulated neutrophils.
